# The Short and Long-Term Effect of Sound Therapy on Visual Attention in Chronic Tinnitus Patients

**DOI:** 10.3390/audiolres12050050

**Published:** 2022-09-13

**Authors:** Mie Laerkegaard Joergensen, Petteri Hyvärinen, Sueli Caporali, Torsten Dau

**Affiliations:** 1 Hearing Systems Section, Department of Health Technology, Technical University of Denmark, 2800 Kgs. Lyngby, Denmark; 2 WS Audiology, 3540 Lynge, Denmark; 3 Department of Signal Processing and Acoustics, Aalto University, 02150 Espoo, Finland

**Keywords:** tinnitus, attention, Attention Network Test (ANT), sound therapy, fractal tones

## Abstract

Sound therapy is one of the most common tinnitus treatments that can be used either to mask or to shift attention away from the tinnitus percept. However, the actual benefit of sound therapy and the mechanisms leading to the benefits remain limited. The objective of this study was to investigate the short-term (15 min) and long-term (2 months) effects of sound therapy on visual attention in chronic tinnitus patients. Visual attention was evaluated with the behavioral Attention Network Task, while the tinnitus-related distress was evaluated with the Tinnitus Handicap Inventory (THI) to quantify the effect of sound therapy. The study included 20 participants with chronic and bothersome tinnitus (>6 months, THI > 18) and 20 matched control participants. All participants took part in a first session consisting of a baseline condition, a short-term sound therapy condition and a silent control condition. The tinnitus participants also took part in a second session that evaluated the long-term effect of the therapy. A reduction in the tinnitus-related distress was found after the long-term use of sound therapy. Furthermore, a reduction in the differential index of the executive control (EC) attention network, indicating improved attention, was found after long-term use of sound therapy in the sound condition but not in the silent control condition. In contrast to earlier research, no differences were found between the tinnitus group and the control group for the baseline measurement of the EC attention network. Overall, the results suggest that there is no link between the visual attention networks and the sound therapy’s effect on tinnitus-related distress.

## 1. Introduction

Tinnitus is the perception of phantom sounds that do not originate from external sources and can severely affect the quality of life of people [1]. People suffering from tinnitus often have a detectable hearing loss [2]. However, the association between hearing loss and tinnitus is complex since only a subgroup of people with hearing loss experience tinnitus and some people without a measurable hearing loss have tinnitus [1]. Despite the non-straightforward relationship between hearing loss and tinnitus, one of the most common treatments has been to restore the missing acoustic stimulation [3]. Sound stimulation can be subdivided into three categories: amplification (hearing aids), sound therapy (sound generator devices) and ‘combination hearing aids’ that provide both amplification and sound therapy. The present study focused only on sound therapy. Sound therapy has been shown to decrease the tinnitus-related distress and increase habituation, reflecting the gradual decrease in an emotional reaction to and awareness of tinnitus [4,5,6,7,8,9]. However, the actual benefit of sound therapy and the mechanisms leading to improvements in tinnitus have remained somewhat elusive. On a general level, it has been postulated that sound therapy may affect cognitive processes such as attention by shifting the attention away from the unpleasant tinnitus perception towards the more pleasant sound therapy [10]. In the present study, the effect of sound therapy on attention in chronic tinnitus patients was investigated.

Attention deficits have previously been studied in tinnitus patients using behavioral paradigms. A number of these studies demonstrated the impact of tinnitus on either selective, sustained or divided attention [11,12,13,14]. Other studies found that tinnitus patients have a reduced executive control ability, i.e., a reduced ability to solve problems such as differentiating between task-relevant and task-irrelevant information [12,15,16]. However, not all studies showed an attentional impairment in tinnitus patients [17,18]. These discrepancies between findings might be explained by the various paradigms that have been employed to investigate attention in tinnitus patients. To overcome this challenge, Heeren et al. [16] demonstrated the need for a systematic and reliable paradigm, where the same test is used to test each sub-component of the attention system. An example of such a paradigm is the Attention Network Test (ANT, [19]) that is based on a validated attention model [20,21]. The model suggests that attention can be subdivided into three independent attentional networks: (1) the ‘alerting network’ that is responsible for maintaining a state of alertness, (2) the ‘orienting network’ that is responsible for shifting attention from one stimulus to another, and (3) the ‘executive control (EC) network’ that is responsible for selecting between task-relevant and task-irrelevant information. The ANT is a visual attention task that measures reaction time (RT) and accuracy to evaluate each of the attention networks and their interaction. Heeren et al. [16] used the ANT to compare the attention networks between a group of tinnitus participants and a group of healthy control subjects. A difference was found in the EC network, suggesting that tinnitus patients were worse at switching their attention between task-relevant and task-irrelevant information.

The focus of the current study was to investigate the behavioral differences in attention before, during and after the use of sound therapy in a group of tinnitus sufferers and to compare the results to those of a control group. Therefore, a visual attention test was selected instead of an auditory one. Furthermore, emphasis was put on an attentional measure that was based on a systematic and reliable attention paradigm that had previously been used to evaluate attention in a tinnitus population [16,18]. The ANT was used to measure the attention networks in a group of chronic tinnitus patients at baseline, after a short-term sound therapy treatment (15 min of listening) and after long-term use of sound therapy (2 months of listening). The baseline and short-term treatment measures were compared to those of a matched control group without tinnitus. Based on results by Heeren et al. [16], it was hypothesized that there would be a difference in the EC between the control group and the tinnitus group at baseline. Furthermore, it was hypothesized that the EC differential index would decrease in the tinnitus group after long-term treatment with sound therapy, i.e., tinnitus patients would improve their abilities to switch attention between task-relevant and task-irrelevant information, and that the decrease in the EC differential index might be linked to the treatment response.

## 2. Materials and Methods

### 2.1. Participants and Sample Size

**Figure 1:**
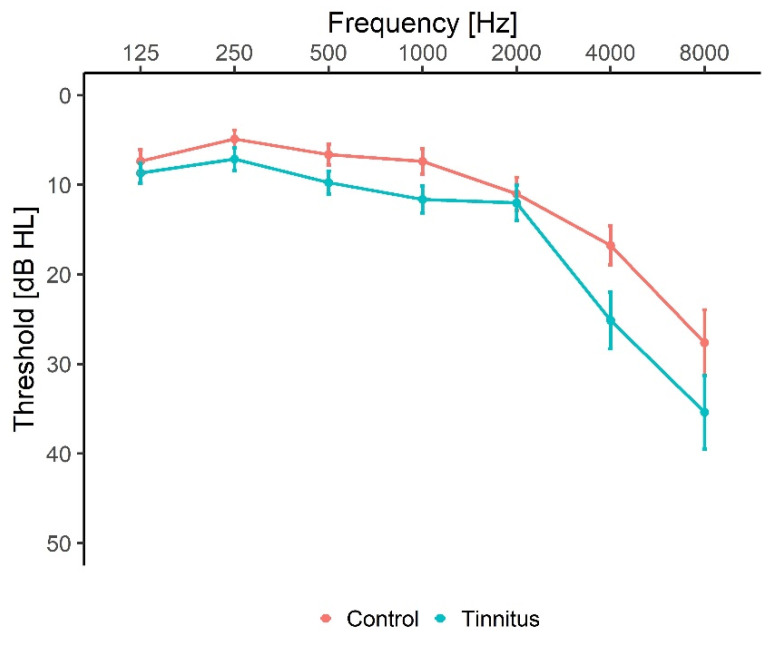
Average audiogram. Hearing thresholds were measured from 125 Hz to 8 kHz. Results were averaged for the left and right ear and shown with mean ± SEM. The red line is the average for the control group, while the blue line is the average threshold for the tinnitus group.

The tinnitus group consisted of 20 participants (14 men, 6 women) aged between 23 and 72 years (51.7 ± 14.2 years) with self-reported tinnitus symptoms. All tinnitus participants were selected based on a hearing screening that assessed both the participants’ audiogram and their tinnitus perception. Prior to inclusion in the study, the participants were asked to fill in the Tinnitus Handicap Inventory (THI) questionnaire and only participants experiencing a negative tinnitus reaction (defined as a THI score above 18) were included in the study. The average Pure Tone Average (PTA) for the tinnitus group was 14.6 dB HL. The control group had 20 participants, matched for age, gender and hearing loss with the tinnitus group (14 men, 6 women, age from 24 to 74 years with an average of 52.0 ± 16.1 years). The average PTA for the control group was 10.5 dB HL. Figure 1 shows the average audiogram for both the tinnitus and control group. The control participants reported no history of chronic tinnitus and experienced spontaneous short-lasting tinnitus less than twice a month.

All participants were recruited from either the local volunteer database at the Hearing Systems Section at the Technical University of Denmark (DTU), from a dedicated trial volunteer website [22] or from the local volunteer database at WS Audiology. The study took place at the Hearing Systems Section at the Department of Health Technology, DTU. The study was approved by the Science-Ethics Committee for the Capital Region of Denmark (reference H-16036391). All of the subjects provided written informed consent before that start of the study.

The sample size was based on the effect size of the EC differential index reported in Heeren et al. [16], where the ANT test was used [16]. They found an effect size of 0.86 when comparing the EC differential index of a control group to the EC differential index of a tinnitus group. Based on this value, the program G*power [23] was used to calculate the sample size of this study. The calculation was based on an independent means *t*-tests for two experimental groups, an alpha level of 0.05 and a power value (1-beta) of 0.8. This yielded a total sample size of 36 participants (18 for each experimental group: tinnitus participants and control participants). The sample size was set more conservatively to 20 participants for each experimental group.

### 2.2. Tinnitus Characteristics in Participants

The description of the percept experienced by the tinnitus participants regarding tinnitus-related distress, loudness, location and percept in the first session before the treatment with sound therapy are summarized in Table 1. The average THI score was mean = 39.3 ± standard deviation (SD) = 3.5 points, which is categorized as a moderate tinnitus handicap. The tinnitus loudness was evaluated on a scale from 0–100 and the average pre-treatment score was mean = 61.6 ± SD = 4.8 points. The majority of the participants (fourteen) described their tinnitus percept as a tone, while three participants (participants 3, 5, and 11) described the percept as noise-like; participant 12 perceived two concurrent tinnitus percepts and participants 6 and 15 were unsure about whether the percept was tonal or noisy. A total of 18 of the 20 participants described their tinnitus as either a high-frequency or a very high-frequency percept. Participant 3 could not identify the tinnitus pitch and participant 12 perceived two tinnitus percepts: one was identified as a high-frequency sound while the other was identified as a low-frequency sound. Moreover, 14 participants described that they could mask their tinnitus perception with either music or TV, however, participants 2 and 8 indicated the percept could not be masked. Four of the participants (5, 14, 19, 20) did not know if their tinnitus could be masked or not.

### 2.3. Hearing Assessment Procedure

Pure-tone audiometry was conducted in a sound-proof booth using a standard clinical audiometer (model AS216, Interacoustics A/S, Middlefart, Denmark) and HD200 headphones (Sennheiser GmbH & Co. KG, Wedermark, Germany) in the frequency range from 125 Hz to 8 kHz.

### 2.4. Questionnaires

Tinnitus severity was evaluated using the standardized outcome questionnaire, the THI [24]. The THI contains 25 questions that can be answered with ‘yes’ (4 points), ‘sometimes’ (2 points) and ‘no’ (0 points). The THI results in a score between 0 and 100 points. Furthermore, patient case history was collected with the Tinnitus Sample Case History Questionnaire (TSCHQ: [25]). The TSCHQ gathers descriptive information about the participants’ tinnitus including tinnitus perception, pitch and loudness.

### 2.5. Sound Therapy

All of the participants were fitted bilaterally with Widex Evoke Passion 440 hearing aids with instant open ear tips. All fittings were performed using the Widex fitting software-Compass GPS (v. 3.4). To avoid the possible effect of amplification as tinnitus treatment, the participants were not compensated for their hearing losses. Instead, all participants were fitted based on a flat audiogram with hearing thresholds of 10 dB at all frequencies from 125 Hz to 8 kHz.

Fractal tones were used as sound therapy in the present study. Fractal tones are harmonic and melodic tones that are unpredictable, but without any sudden changes in tonality and tempo. The fractal tones are randomly generated by a patented algorithm in the hearing aids. A random number sequence is generated by the fractal generator. These numbers are assigned their pitch, tempo, and intensity based on predefined rules. In the current study, the standard parameters from the Compass GPS fitting software were used for all participants. Up to 12 music generators decode these numbers and generate the desired fractal tone. Moreover, the melodic structures are based on a pseudorandom signal, generated by a maximum length sequence generator. The fractal tones were selected with a custom-made MATLAB script to ensure that the program with the fractal tones was the only available program in the hearing aids. The participants were asked to evaluate the pleasantness of the individual fractal tones and choose the one they preferred. The participants could choose between 5 fractal tone types (‘aqua’, ‘coral’, ‘green’, ‘lavender’ and ‘sand’). The preferred fractal tone was used for both the short-term and long-term treatment. The average pleasantness for the tinnitus participants were 8.0 ± 1.2 points (mean ± standard deviation on a scale from 0–10). The participants were guided to make sure that the fractal tones were audible when they were used, but that they should not interfere with conversational speech. All of the participants were given a remote control to adjust the level of the fractal tones to ensure that the therapy could be used comfortably in different environments. The participants were asked to use the sound therapy for at least 3–4 h per day. Datalog data from the hearing aids showed that the hearing aids had on average been active for 6.0 ± 4.4 h per day (mean ± standard deviation, data from 18 participants due to missing data: Two participants lost their hearing aids during the study. These hearing aids were immediately replaced with new ones, but the datalogs could not be retrieved from the lost hearing aids).

### 2.6. Behavioural Data

**Figure 2:**
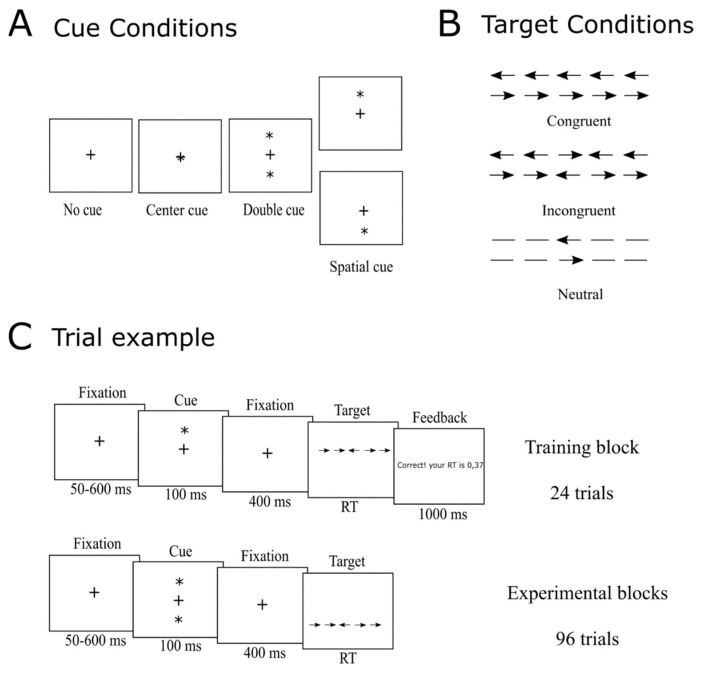
Overview of the ANT. (**A**) Cue conditions. (**B**) Target conditions. (**C**) Example of a training block trial (first row) and an experimental block trial (second row). * = cue, + = fixation cross, ← = arrow pointing left, → = arrow pointing right. Adapted from [19] ©2002 by the Massachusetts Institute of Technology. All rights reserved.

The Attention Network Test (ANT) was used to evaluate the three independent attention networks: alerting, orienting and executive control [19]. The participants were asked to select the [1direction of a target arrow seen on a screen as fast and accurately as possible by pressing the corresponding button (left or right) on a response pad. The target arrow was shown in the middle of a horizontal line that appeared either on the top or at the bottom of the screen. The horizontal line consisted of the target arrow and two flankers on each side of the target arrow. There were three different target conditions: (1) ‘congruent’ where the flankers were arrows pointing in the same direction as the target arrow; (2) ‘incongruent’, where the flankers were arrows pointing in the opposite direction of the target arrow; and (3) ‘neutral’ where the flankers were lines. An overview of the target conditions is shown in Figure 2B. The targets were preceded by a visual cue and there were four different types of cue conditions (Figure 2A): ‘no cue’, ‘center cue’ (the asterisk appeared at the same place as the fixation cross), ‘double cue’ (an asterisks appeared both above and below the fixation cross) and ‘spatial cue’ (an asterisk appeared either above or below the fixation cross indicating where the upcoming target would appear). Each trial had the following structure (Figure 2C): (1) a white central fixation cross was shown on a black screen (50–600 ms); (2) a cue appeared (for 100 ms); (3) a fixation cross appeared (for 400 ms); (4) a horizontal line with the target and flankers appeared either above or below the fixation cross (the target remained on the screen until the participant responded); and (5) a fixation cross appeared again (400 ms). The RT (time between the onset of the target and the response) and the accuracy of the response were recorded for each trial. Please find the detailed description of the RT recordings below. Each ANT consisted of 288 trials that were divided into three rounds of 96 trials each and with short breaks between each round. Each round consisted of two repetitions of the 48 possible trial combinations: four cues (no cue, central cue, double cue and spatial cue), three flanker conditions (congruent, incongruent and neutral), two directions of the target arrow (left or right) and two locations (top or bottom of the screen). The trial combinations were presented in random order within each round. The task was programmed and presented using PsychoPy [26]. The task took place in a sound-proof listening booth and lasted approximately 15 min.

The differential indices of each attention network were calculated for both the RTs and the accuracy. The differential indices of the alerting network: mean RT_No Cue_mean—RT_Double Cue_ and mean accuracy _No Cue_—mean accuracy _Double Cue_. The differential indices of the orienting network: mean RT_Central Cue_—mean RT_Spatial Cue_ and mean accuracy _Central Cue_—mean accuracy _Spatial Cue_. While the differential indices of the executive control network included: mean RT_Incongruent_—mean RT_Congruent_ and mean accuracy _Incongruent_—mean accuracy _Congruent_.

### 2.7. RT Measure

Responses for the ANT task were collected using a custom-made response button box. Button presses were converted into transistor-transistor logic (TTL) pulses using an Arduino Uno microcontroller board and recorded with a BioSemi ActiveTwo EEG system. Onset timing of the trial was identified using an optical sensor (g.TRIGbox accessory) attached to the screen. During the presentation of the visual stimulus, a white rectangle was drawn in the corner of the screen where the sensor was attached. At other times, the rectangle was black. The output of the optical sensor was also coded as a TTL signal and connected to the EEG recording equipment. This allowed a millisecond-precise measurement of the RTs. A specialized response button setup was chosen because of timing uncertainty related to standard computer peripherals such as USB keyboards and mice [27]. Additional variable delays of 20–70 ms can be introduced by non-specialized equipment, which may be detrimental for the data quality in RT experiments [27]. To ease the analysis of responses, the Arduino board served a double purpose by also emulating a USB keyboard. The emulated keypress events were used for controlling the PsychoPy experiment.

Inspection of the differences between the calculated RTs based on the EEG triggers and the ones calculated based on the Psychopy data showed that the difference varied from trial to trial between 50 ms and 500 ms. Due to the inconsistency in the RTs calculated from the Psychopy data, the statistical analyses were only performed on the EEG trigger data to assure the accuracy of the RTs.

### 2.8. Procedure

In the first session, all of the participants were given a thorough introduction to the aims of the study and the included methods before they were asked to provide written informed consent. Hearing aids were then fitted with the GPS fitting software and a customized MATLAB script. The customized MATLAB script was used to remove the standard universal program of the hearing aids, to make sure that the participants could only use the hearing aids to play the sound therapy. Each fractal tone was evaluated by the participants who were asked to select the most pleasant fractal tone. This sound was then used for the remaining part of the study.

**Figure 3:**
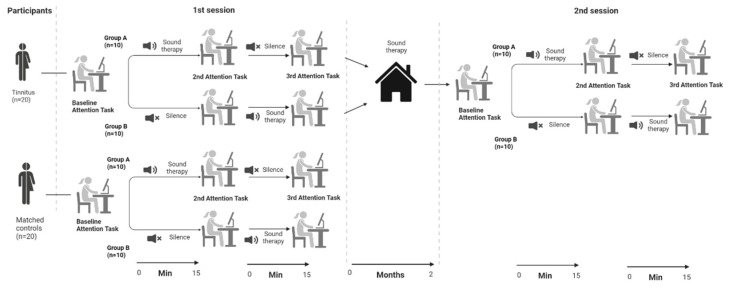
Overview of the study. Created with [28].

An overview of the study procedure can be found in Figure 3. Before the beginning of the ANT, the participants were asked to read the instructions on the computer screen. The instructions were clarified by the experimenter and a training session started that consisted of 24 randomly selected trials. Any remaining questions were answered by the experimenter before the beginning of the task. After the training session, the participants performed the baseline ANT. After the baseline measurement, the participants were randomly allocated into one of two groups. The participants in group A listened to 15 min of sound therapy before they started the ANT for the second time. The sound therapy was still playing while they conducted the ANT. The participants in group A then had 15 min to relax in silence before they started the ANT for the final time. The participants in group B started with the 15 min relaxation in silence that was followed by the second ANT. Afterwards they received 15 min of sound therapy before they conducted the final ANT (while listening to the sound therapy). After the third ANT task was finished, the control participants were debriefed, while the tinnitus participants received instructions on how to use the hearing aids.

The tinnitus participants were asked to use the sound therapy on a daily basis for at least 3–4 h a day. After two months of sound therapy use, the tinnitus participants took part in a second session. In the second session, the participants filled in the THI questionnaire before they conducted the three ANTs in the same order as described above for the first session. The second sessions were planned to take place at the same time of the day ± 2 h as the first session, to limit the effects of the circadian rhythms on the attention paradigm.

### 2.9. Statistical Analysis

Welch′s *t*-tests were used to compare the difference scores for RT and accuracy for each attentional network separately for each condition. Bonferroni adjusted *p*-values were used to correct for multiple comparisons for each attentional network separately. A Pearson’s correlation was used to compare the correlation between the EC difference and the THI difference. The data from 20 tinnitus participants and 20 control participants were included in the analysis.

## 3. Results

### 3.1. Session 1: The Influence of Tinnitus on Attention Networks

**Figure 4:**
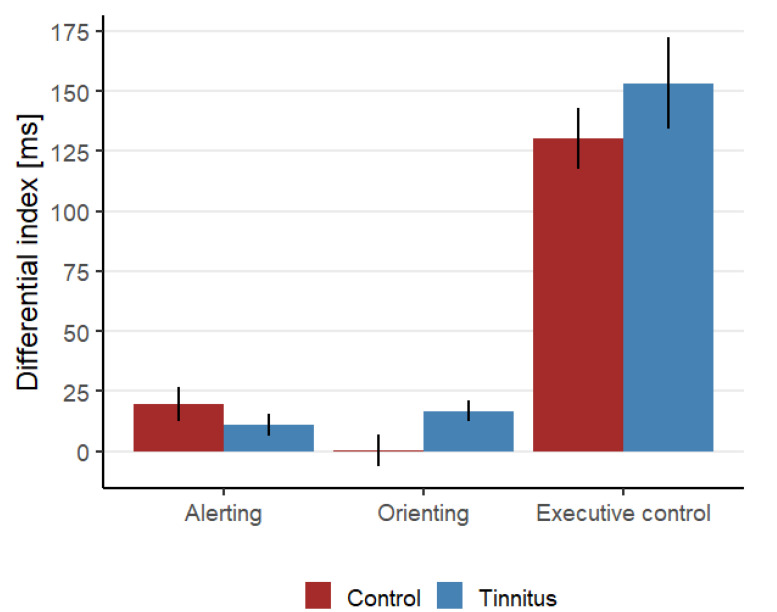
The differential indexes calculated based on RTs for the alerting (RT_No Cue_—RT_Double Cue_), orienting (RT_Central Cue_—RT_Spatial Cue_) and executive control (RT_Incongruent_—RT_Congruent_) attention networks for both the control group and the tinnitus group at baseline. Error bars represent standard errors of the mean.

The overall task accuracy was very high both for the control group (98.2%, SD = 3.1%) and the tinnitus group (session 1: 98.2%, SD = 2.6%; session 2: 98.1%, SD = 2.6%). All of the following analyses were performed on difference scores for RT and accuracy for each attentional network separately. Figure 4 shows an overview of the differential indexes for the RT for each attention network at baseline. To evaluate if tinnitus affects the attention networks, the baseline ANT measurements from the tinnitus and control groups from the first session were compared.

**Executive Control.** A comparison between the control group’s and the tinnitus group’s RT differential index did not show any significant differences between the groups (t(31.1) = −1.01, *p* = 0.32), neither was there any difference in the accuracies between the two groups (t(35.78) = 0.69, *p* = 0.49).

**Alerting and orienting.** No statistically significant differences were found for the RT and accuracy differential indexes. An overview of the statistical comparisons can be found in Table 2 (top section).

### 3.2. Session 1: Short-Term Effect of Sound Therapy on Attentional Networks

To evaluate the effect of short-term sound therapy on the attention networks, the silent and sound ANT measurements from the tinnitus group from the first session were compared.

**Executive Control.** The average RT differential index was found to be significantly higher in the sound condition (148.1 ± 15.7 ms) than in the silent condition (136.0 ± 16.4 ms, t(17) = −2.40, *p* = 0.02826). However, no statistically significant differences were found in the average accuracy for the two conditions (t(17) = −1.22, *p* = 0.24). This finding suggests that the sound therapy initially reduces the participant’s ability to differentiate between task-relevant and task-irrelevant information.

**Alerting and orienting.** No statistically significant differences were found for the RT and accuracy differential indexes. An overview of the statistical comparisons can be found in Table 2 (middle section).

### 3.3. Session 2: Long-Term Effect of Sound Therapy on Attention Networks for Chronic Tinnitus Patients

To evaluate the effect of long-term sound therapy on the attention networks for tinnitus sufferers, the silent and sound ANT measurements from the tinnitus groups from the first and second session were compared. To correct for the two condition comparisons, the *p*-value was Bonferroni adjusted from 0.5 to 0.025.

**Figure 5:**
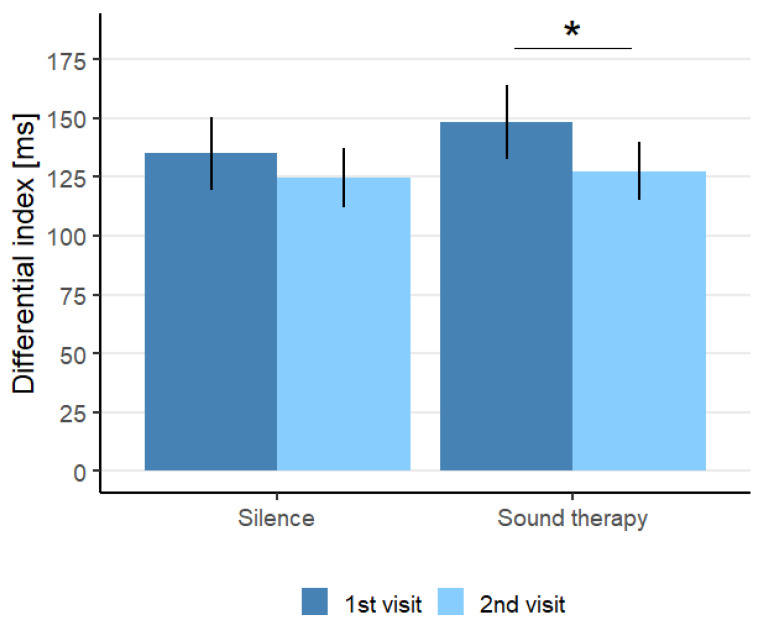
The differential indexes calculated based on RTs for the executive control attention networks for the tinnitus group in first visit (dark blue) and second visit (light blue) in both the silent and ST conditions. * *p* < 0.05. Error bars represent standard errors of the mean.

**Executive Control.** The average RT differential index was found to be significantly lower in the second session (127.5 ± 12.4 ms) than in the first session (148.1 ± 15.7 ms, t(17) = 2.66, *p* = 0.01654) in the sound condition. However, no statistically significant differences were found in the average RT differential index for the two sessions (t(18) = 1.32, *p* = 0.20) in the silent conditions. Figure 5 shows the average RT differential indices for both the sound and silent condition. No statistically significant differences were found for the accuracy differences in the sound condition (t(17) = 1.73, *p* = 0.10) or in the silent condition (t(17) = 0.52, *p* = 0.61).

**Alerting and orienting.** No statistically significant differences were found for the RT and accuracy differential indexes. An overview of the statistical comparisons can be found in Table 2 (bottom section).

### 3.4. Session 2: Effect of Sound Therapy on Tinnitus-Related Distress

**Figure 6:**
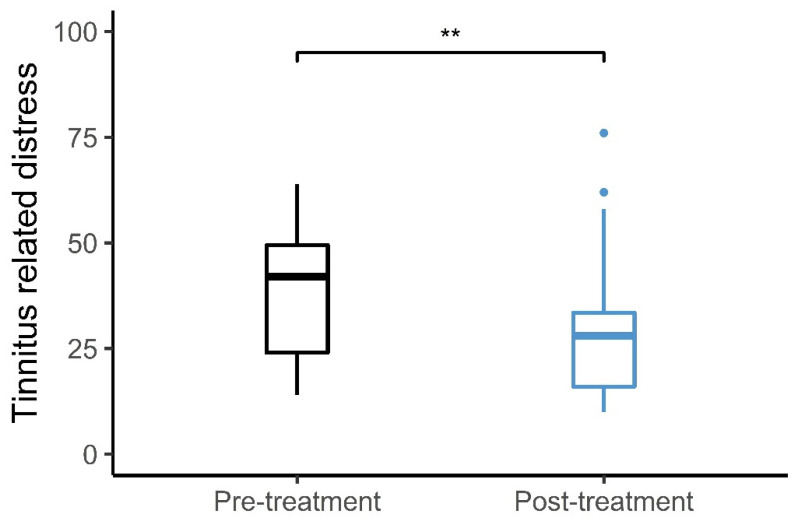
Tinnitus distress. Average THI scores pre- and post- long-term treatment with sound therapy. ** *p* < 0.01.

Figure 6 shows the average change in the THI scores from pre- to post-sound therapy treatment. The average pre-treatment THI score was 39.3 ± 3.5 points, while the average post-treatment THI score was 30.1 ± 4.0 points. The average change in the THI score after the use of sound therapy was 9.2 ± 2.4 points. Sixteen participants experienced a decrease in the THI score, while two participants indicated the same THI score and two participants experienced an increase in the THI score after the treatment. Of the 16 participants experiencing reductions in THI scores, 9 experienced clinically significant reductions. The clinically significant reduction was defined as an improvement of more than 7 points as suggested by Zeman et al. [29]. There was a statistically significant difference between the average pre-treatment THI scores and the post-treatment THI scores (t(19) = 3.84, *p* = 0.0011).

### 3.5. Correlation between THI and EC Improvements

To evaluate whether the improvement in tinnitus distress was related to the improvements in the EC attention network, the differences of the THI score between pre- and post-treatment were correlated with the difference in the differential index of the EC network in the sound condition. No statistically significant correlation was found between the differences in THI scores and the differences in the differential index for the EC network (R = −0.37, *p* = 0.14). Figure S1 shows the correlation between the differences in the THI scores and the differences in the EC attention network between pre- and post-treatment.

## 4. Discussion

The present study evaluated the effect of short- and long-term sound therapy on the tinnitus-related distress and the EC attention network in chronic tinnitus patients. The study investigated two hypotheses: (1) Tinnitus patients have a higher differential index for the EC network compared to healthy controls, i.e., tinnitus patients are less capable at switching attention between task-relevant and task-irrelevant information than healthy controls. The results of the present study did not support this hypothesis. No statistically significant difference was found between the control and the tinnitus group when comparing the RT and accuracy differential index for the EC network. (2) Sound therapy leads to reductions in the tinnitus-related distress by switching attention away from the tinnitus percept and towards a more pleasant sound. The present study found a reduction in the tinnitus-related distress after long-term use of sound therapy. Furthermore, a reduction was found in the EC differential index for the sound condition, but not for the silent condition. However, no correlation was found between the reduction in tinnitus-related distress and the reduction in the EC differential index.

Similar to previous findings [30,31], the present study showed a significant decrease in the tinnitus-related distress after long-term use of sound therapy. Large individual differences were found regarding the effect of the long-term sound therapy treatment. Of the 20 tinnitus participants included in the study, 9 (i.e., 45% of the participants) experienced a clinical reduction in the tinnitus distress. Hobson et al. [32] showed that the therapeutic effect of sound therapy varies from 20% to 80%, so the results of the present study are consistent with previous findings. Furthermore, in accordance with previous findings, the present study found an improvement in terms of attention ability after long-term use of sound therapy [7,9]. However, the statistically significant difference between the first and the second session was only observed in the sound condition and not in the silent condition. It is therefore possible that the difference found in the sound condition does not represent a general improvement in attention but demonstrates that the tinnitus participants have grown accustomed to the sound therapy during the two months use. This assumption is based on the fact that the differential index was larger in the sound condition than in the silent and baseline conditions for the first session, suggesting that the sounds initially worsened the attention. In the second session, there were no differences between the differential indices in the sound and silent conditions. Furthermore, no correlation was found between the reduction in tinnitus-related distress and the improvement in the differential index of the EC attention network. Thus, although long-term use of sound therapy had a small influence on attention as measured by the ANT, it seems that the main effect of sound therapy on tinnitus-related distress was not mediated by improvements in attention.

In contrast to previous findings, the present study did not demonstrate an impairment in the EC for the tinnitus group compared to the matched control group [11,15,16]. However, the results were consistent with the results from Jensen et al. [18], who also did not report an impairment of the EC attention network in tinnitus patients. Although, the studies by Stevens et al. [11] and Andersson et al. [15] used different tests to investigate the attention in tinnitus patients, the present study used the same test as Heeren et al. [16] and the lack of replication of previous findings, is therefore not thought to be caused by differences in the used attention test. However, there were other differences in the study design that might explain the differences in the results across the studies. In the present study, the control group was matched based on hearing thresholds, in addition to gender and age, while hearing thresholds were not considered in the studies by Heeren et al. [16] and Andersson et al. [15]. Stevens et al. [11] did report hearing thresholds but there was a 10 dB difference in the PTA between the tinnitus and control groups (tinnitus group had a worse hearing). In the ANT, the target stimuli are presented visually and the effect of hearing impairment on visual attention is still limited. However, a study suggested that hearing loss was associated with increased RTs during a visual sustained attention task [33]. It is therefore possible that the selection of the control group can explain the inconsistent results. This is supported by a recent meta-analysis that did not find a difference in attention abilities between a tinnitus group and a control group when hearing thresholds were matched [34].

The present study showed statistically significant improvements in tinnitus distress following the use of sound therapy for two months in chronic tinnitus patients. However, no tinnitus control group was included in the study to compare the sound therapy treatment with another treatment or a waiting list condition. A tinnitus control group was not included because the purpose of the present study was not to evaluate the effect of the sound therapy as a tinnitus treatment, but to investigate if the effect of the sound therapy could be explained by an impact on the attention in tinnitus patients. Furthermore, the THI questionnaire was used in the present study to evaluate the effect of the long-term sound therapy treatment. The THI questionnaire is widely used to evaluate tinnitus-related distress and has been shown to have great reliability and validity [24,35]. However, the THI was not developed to evaluate a tinnitus treatment such as, e.g., the Tinnitus Functional Index (TFI). The present study matched the tinnitus and control groups with regards to gender, age and hearing thresholds. However, other factors can impact both attention and the tinnitus percept. Several previous studies have shown that cognitive impairment, anxiety, depression and insomnia can impair attention abilities [36,37,38,39]. These factors were not evaluated in the present study, and it is therefore possible that there were differences between the control and tinnitus groups that might have affected the attention abilities measured. Furthermore, tinnitus often co-occurs with hyperacusis, and sound therapy has been used as a common treatment of hyperacusis [40,41,42]. In the present study, hyperacusis was not evaluated so it is not known if the sound therapy affected the hyperacusis. Future studies may benefit from including measures of hyperacusis, cognition, anxiety, depression and insomnia when comparing tinnitus and control groups. Furthermore, tinnitogenic conditions and medications were not thoroughly examined in the tinnitus group, and it is therefore not possible to evaluate whether they might have influenced the observed results.

Although the present study suggests that long-term use of sound therapy is a beneficial tinnitus treatment for patients suffering from tinnitus-related distress, it also demonstrates large individual differences in the effects of the treatment. This indicates that the sound therapy treatment may be further improved and individualized, or that sound therapy is only beneficial for a subgroup of tinnitus patients. More information is still needed about how tinnitus patients can be divided into subgroups. The present study showed that improvements in the tinnitus-related distress are not correlated with reductions in any of the visual attention networks. This suggests that other mechanisms underlie the beneficial effects of sound therapy on the tinnitus-related distress. Future studies may therefore focus on the link between the tinnitus-related distress and other cognitive processes.

## Figures and Tables

**Table 1:** Description of tinnitus percept based on answers from the Tinnitus Sample Case History Questionnaire. Tinnitus location: BE = Both ears equally, BE(R) = Both ears, mainly the right ear, BE(L) = Both ears, mainly the left ear, LE = Left ear, RE = Right ear, IH = Inside head. Tinnitus pitch: VHF = Very High-frequency, HF = High-frequency, MF = Medium frequency, LF = Low-frequency.

Participant	Tinnitus Location	Tinnitus Loudness	Tinnitus Percept	Tinnitus Pitch	Tinnitus Masking	Pre-THI	Post-THI
**1**	BE	100	Tone	VHF	Yes	60	62
**2**	IH	70	Tone	HF	No	58	32
**3**	BE (L)/IH	65	Noise	Don’t know	Yes	38	16
**4**	IH	40	Tone	VHF	Yes	44	28
**5**	LE	75	Noise	HF	Don’t know	64	76
**6**	BE (R)	15	Tone/Noise	VHF	Yes	44	42
**7**	LE/BE (L)	20	Tone	HF	Yes	18	10
**8**	BE/IH	70	Tone	VHF	No	62	58
**9**	BE	75	Tone	VHF	Yes	30	28
**10**	BE	57.5	Tone	VHF/HF	Yes	48	16
**11**	BE	30	Noise	HF	Yes	22	16
12 *	RE	70	Tone/noise	HF/LF	Yes	46	32
**13**	IH	85	Tone	VHF	Yes	54	38
**14**	BE (R)	70	Tone	HF	Don’t know	14 **	12
**15**	IH	65	Noise/tone	HF	Yes	24	24
**16**	RE	60	Tone	HF	Yes	18	18
**17**	IH	60	Tone	HF	Yes	34	30
**18**	LE	50	Tone	HF	Yes	40	20
**19**	BE (R)/IH	75	Tone	HF	Don’t know	44	32
**20**	BE	80	Tone	VHF	Don’t know	24	12

* Perceives two different sounds. ** On day of prescreening the THI-score was >18 and participant was therefore included in study. However, during first session a THI score of 14 was measured.

**Table 2:** Overview of the statistical comparisons for the alerting (Alert), orienting (Orient) and executive control (EC) networks.

Comparison	Network	Condition	Statistics	*p*-Value
*The influence of tinnitus on attention networks*				
Baseline control group × baseline tinnitus group	Alert	RT	t(31.1) = 1.02	*p* = 0.32
		Accuracy	t(32.5) = −1.69	*p* = 0.10
	Orient	RT	t(30.1) = −2.04	*p* = 0.050
		Accuracy	t(29.4) = 1.88	*p* = 0.070
	EC	RT	t(31.1) = −1.01	*p* = 0.32
		Accuracy	t(35.78) = 0.69	*p* = 0.49
*Short-term effect of sound therapy on attentional networks*				
Short-term sound tinnitus group × short-term silent tinnitus group	Alert	RT	t(17) = 0.36	*p* = 0.72
		Accuracy	t(17) = 0.22	*p* = 0.83
	Orient	RT	t(17) = −1.15	*p* = 0.26
		Accuracy	t(17) = −0.42	*p* = 0.68
	EC	RT	t(17) = −2.40	*p* = 0.028
		Accuracy	t(17) = −1.22	*p* = 0.24
*Long-term effect of sound therapy on attention networks for chronic tinnitus patients*				
Short-term silent tinnitus group × long-term silent tinnitus group	Alert	RT	t(17) = −0.59	*p* = 0.56
		Accuracy	t(17) = −1.72	*p* = 0.10
	Orient	RT	t(17) = 0.04	*p* = 0.97
		Accuracy	t(17) = −0.72	*p* = 0.48
	EC	RT	t(18) = 1.32	*p* = 0.20
		Accuracy	t(17) = 0.52	*p* = 0.61
Short-term sound tinnitus group × long-term sound tinnitus group	Alert	RT	t(17) = −0.41	*p* = 0.69
		Accuracy	t(17) = −0.13	*p* = 0.90
	Orient	RT	t(17) = −1.11	*p* = 0.28
		Accuracy	t(17) = 0.076	*p* = 0.94
	EC	RT	t(17) = 2.66	*p* = 0.017
		Accuracy	t(17) = 1.73	*p* = 0.10

## Data Availability

The data presented in this study is available on request from the corresponding author.

## Supplementary Materials

The following supporting information can be downloaded at: https://www.mdpi.com/article/10.3390/audiolres12050050/s1.
