# Supplementary figures and images for: The Short and Long-Term Effect of Sound Therapy on Visual Attention in Chronic Tinnitus Patients
Source: Audiol Res. 2022 Sep 13;12(5):493–507. doi: 10.3390/audiolres12050050 (PMC9498397; doi:10.3390/audiolres12050050)

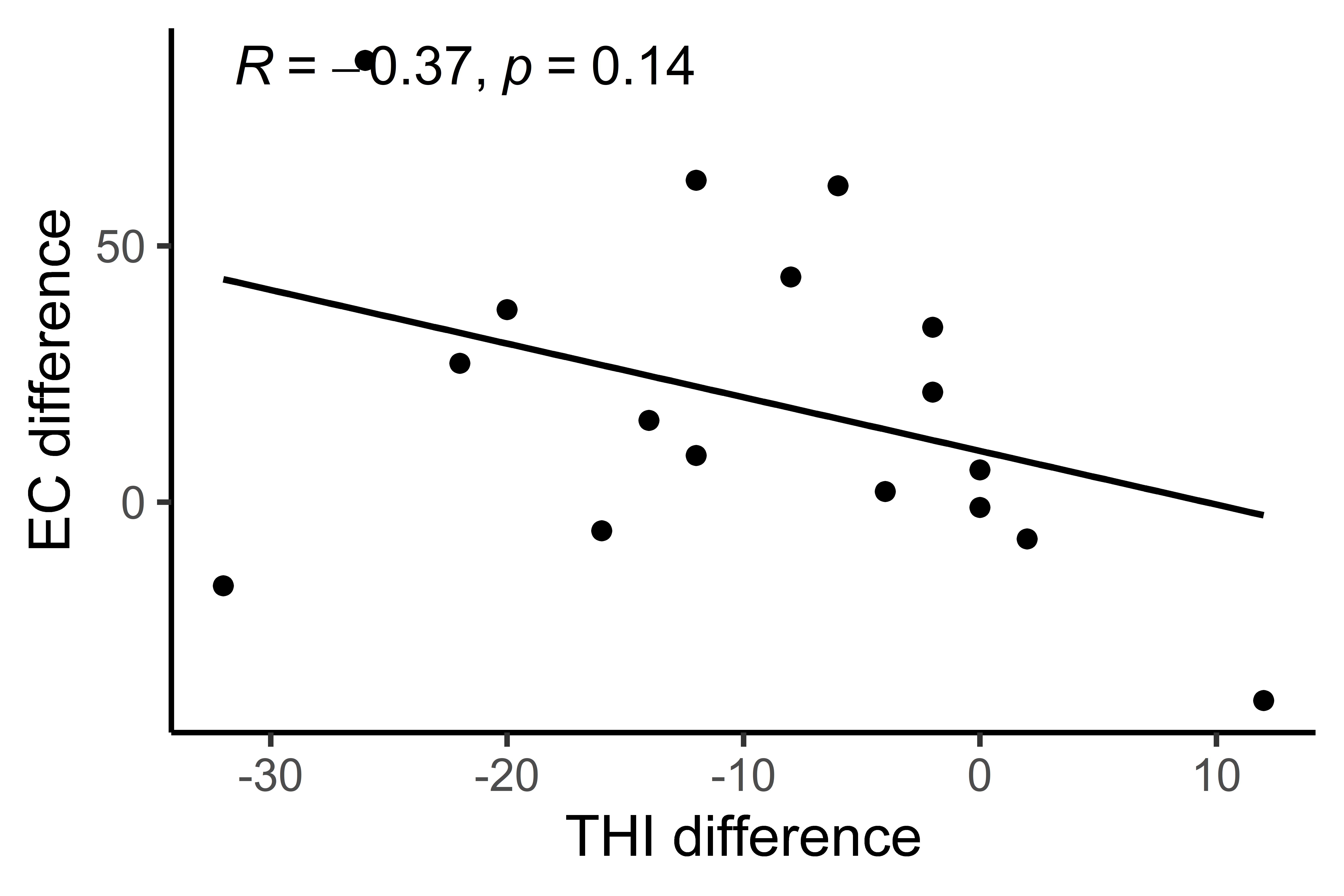

Supplement: Supplementary file 1 [file audiolres-12-00050-s001.zip › audiolres-1839070-supplementary.jpeg]
